# Revealing the Influence of the Degumming Process in the Properties of Silk Fibroin Nanoparticles

**DOI:** 10.3390/polym11122045

**Published:** 2019-12-09

**Authors:** Guzmán Carissimi, A. Abel Lozano-Pérez, Mercedes G. Montalbán, Salvador D. Aznar-Cervantes, José Luis Cenis, Gloria Víllora

**Affiliations:** 1 Department of Chemical Engineering, Faculty of Chemistry, University of Murcia (UMU), Campus de Espinardo, 30100 Murcia, Spain; guzmancarissimi@gmail.com; 2 Department of Biotechnology, Instituto Murciano de Investigación y Desarrollo Agrario y Alimentario (IMIDA), La Alberca, 30150 Murcia, Spain; abel@um.es (A.A.L.-P.); sdac1@um.es (S.D.A.-C.); josel.cenis@carm.es (J.L.C.); 3 Department of Chemical Engineering, University of Alicante, Apartado 99, 03080 Alicante, Spain; mercedes.garcia@um.es

**Keywords:** silk fibroin nanoparticles, cocoon degumming, *Bombyx mori*, ionic liquids, ultrasound, autoclave

## Abstract

Several studies have stated that the process used for sericin removal, or degumming, from silk cocoons has a strong impact in the silk fibroin integrity and consequently in their mechanical or biochemical properties after processing it into several biomaterials (e.g. fibers, films or scaffolds) but still, there is a lack of information of the impact on the features of silk nanoparticles. In this work, silk cocoons were degummed following four standard methods: autoclaving, short alkaline (Na_2_CO_3_) boiling, long alkaline (Na_2_CO_3_) boiling and ultrasounds. The resultant silk fibroin fibers were dissolved in the ionic liquid 1-ethyl-3-methylimidazolium acetate and used for nanoparticle synthesis by rapid desolvation in polar organic solvents. The relative efficiencies of the degumming processes and the integrity of the resulting fibroin fibers obtained were analyzed by mass loss, optical microscopy, thermogravimetric analysis, infrared spectroscopy and SDS-PAGE. Particle sizes and morphology were analyzed by Dynamic Light Scattering and Field Emission Scanning Electronic Microscopy. The results showed that the different treatments had a remarkable impact on the integrity of the silk fibroin chains, as confirmed by gel electrophoresis, which can be correlated with particle mean size and size distribution changes. The smallest nanoparticles (156 ± 3 nm) and the most negative Z potential (−30.2 ± 1.8 mV) were obtained with the combination of long treatment (2 h) of boiling in alkaline solution (Na_2_CO_3_ 0.02 eq/L). The study confirms that parameters of the process, such as composition of the solution and time of the degumming step, must be controlled in order to reach an optimum reproducibility of the nanoparticle production.

## 1. Introduction

For most medical applications of silk biomaterials [1], silk fibroin (SF) must be efficiently purified from silk cocoons (SC) by removing the silk sericin (SS) by means of the process known as degumming, and this key step is commonly recognized as one of the most important procedures in the production of silk-based materials [2]. This purified SF can be dissolved in solutions, such as LiBr 9.3 mol/L [3] or Ajisawa’s reagent (CaCl_2_/EtOH/H_2_O, 1:2:8 in molar ratio) [4], the most commonly used, but also in some ionic liquids, such as 1-butyl-3-methylimidazolium chloride ([bmim^+^][Cl^−^]) [5,6] or 1-ethyl-3-methylimidazolium acetate ([emim^+^][AcO^−^]) [7], which are recognized as efficient alternatives due to their interesting properties as green solvents [8,9,10]. Ionic liquids, with a halogen or a small carboxylate anion, are capable of breaking the network of intra- and intermolecular hydrogen bonds and thus dispersing the peptide chains of the SF [5]. The conventional dissolution of SF in highly concentrated salts is a time-consuming multistep process that involves dialysis against distilled water for several days in order to remove the salts and obtain an aqueous solution of SF [3] prior to their regeneration in solid state in the form of SF nanoparticles (SFN) [4]. Furthermore, the aqueous SF solution obtained after dialysis is relatively unstable at 4 °C, evolving to a hydrogel due to changes in the pH of the solution or a change in pressure or shaking [11,12]. Alternatively, the ionic liquid-SF solutions can be precipitated directly into nanoparticles [6,7] avoiding the dialysis step and giving more stable SF solutions.

Silk sericin comprises a water-soluble globular protein family that are soluble in hot or boiling water due to their amino acid composition, with an abundance of mostly polar amino acids such as serine or aspartic acid [13]. The degumming process can be performed using different methodologies that are based on the differences in solubility between both SS and SF [14,15,16,17]. In general, a hot aqueous medium is used as extracting medium, preferably alkaline, in which SS is soluble and, thus, is separated from SF fibers, which remain essentially insoluble under these conditions. Several degumming methods have been published recently, showing the effect of this critical processing step on the structure and properties of SF for multiple applications [18,19,20,21,22,23,24,25]. Among the different methods, the use of an autoclave in different configurations is frequently mentioned: in an aqueous solution subjected to 121 °C for 20 min [21] or using water vapor generated in the autoclave [16]. Although this method presents a lower yield than the protocols that use alkaline boiling, it has the advantage of lower production costs while maintaining acceptable mechanical characteristics of SF fibers and is very useful when trying to recover salt-free SS by spray-drying [26,27]. A frequently used degumming method for biomedical applications of silk fibroin is that involving boiling the cocoons in alkaline solution (Na_2_CO_3_ 0.02 mol/L) for 30 min [3]. This method extracts SS very well and involves low SF degradation, which makes it useful for applications where high purity and strong integrity of SF is needed. Other methods have also been described in which boiling is used for longer times, e.g. 120 min [28], or by extraction in hot water assisted by ultrasound [19] or using a solution of 8 mol/L urea at 80 °C for 120 min [21]. Also, experimental works on the removal of SS by different proteases enzymes [14,29] or organic acids, such as tartaric acid, have also been seen to perform efficiently [30,31].

Previous studies have found that different degumming processes affect SF yarns properties [15,19,21,32,33] and also concern the production of SF-based engineered materials like films [34,35,36], 3D scaffolds [28], or electro-spun mats [37,38]. Several studies have stated that the process used for silk cocoon degumming has a strong impact in the silk fibroin integrity and their mechanical properties after processing it into biomaterials, but still, there is a lack of information of the impact of this process on the obtained silk nanoparticles. Only few partial studies have been published on how the degumming affects particle formation but have been focused on microparticles or have used only one degumming method for preparation of the nanoparticles. For example, Wang et al. have studied the effect on the preparation of silk fibroin microspheres, stating that the degumming method (Na_2_CO_3_ 30′ or urea 8 mol/L) has a great effect on the microparticle size distribution, through the changes on the molecular weight of the fibroin [34]. Seib et al. reported the effect of degumming time (5, 20, and 60 min) on nanoparticle formation concluding that only the silk degummed for 60 min (boiling time) was able to efficiently produce nanoparticles by desolvation with acetone [39]. The authors recognize that is, therefore, unknown how boiling time impacts particle features that would affect the efficiency of SFN as drug delivery system.

Numerous research studies have shown the excellent characteristics of SFNs as a vehicle for drugs and biomolecules [40,41,42,43,44], so their scalable production for commercial purposes would be considered in coming years. For this reason, it is of great interest to know all the parameters that affect the formation of nanoparticles to standardize the process and control their features.

The present study focuses on how the degumming process affects SFNs structural features (morphology, size distribution, Z-potential, and surface charge density), intending to delve into the effects of all the stages of the silk processing in the properties of the biomaterials, specifically focused on the reproducibility of the processes [6,12,38,45]. In order to assess the different silk degumming processes and their possible effect on SFNs, silk was degummed by means of four different methods, namely autoclaving (D1), alkaline treatment with Na_2_CO_3_ for 30 min (D2) or 120 min (D3), and a high-power ultrasound treatment (D4). Each SF obtained by the mentioned degumming methods was used to prepare SFN. The effectiveness of the degumming process was assessed by the weight loss. Protein integrity after the different degumming processes and dissolution in ionic liquid was studied by Sodium Dodecyl Sulfate-Polyacrylamide Gel Electrophoresis (SDS-PAGE). A morphological observation of SF fibers after the degumming processes was made by Optical Microscopy and Scanning Electron Microscopy (SEM). The thermal stability of the SF fibers was measured by Thermogravimetric Analysis (TGA-DTA). The changes in the secondary structure of the protein were monitored by the crystallinity index (CI%) of the sample obtained from the Fourier Transformed Infrared Spectroscopy (FTIR) Analysis. Finally, the morphology, size distribution, Z-potential, and surface charge density of the obtained nanoparticles were analyzed by Field Emission Scanning Electron Microscopy (FE-SEM) and Dynamic Light Scattering (DLS), respectively.

## 2. Materials and Methods

### 2.1. Materials

Silk cocoons (SC) were obtained from silkworms *Bombyx mori* reared in the sericulture facilities of IMIDA (Murcia, Spain) and raised on a diet of fresh, natural *Morus alba L*. leaves. Cocoons were then stifled to kill the pupae by means of dry heat (85 °C) [45]. The intact chrysalides were extracted manually from the cocoons prior to silk processing. All reagents and solvents were purchased from Sigma-Aldrich (Madrid, Spain) with the exception of methanol (Honeywell, Seelze, Germany) and 1-ethyl-3-methylimidazolium acetate ([emim^+^][AcO^−^]) 95% (Iolitec, Heilbronn, Germany).

### 2.2. Degumming Methods

In this work, four degumming methods were chosen among the most representative of the commonly used in literature and were named as: D1) Degumming by autoclave [21]; D2) Degumming by short alkaline boiling (30 min) [3,38]; D3) Degumming by long alkaline boiling (120 min) [28]; D4) Ultrasonication with probe in water [19]. A brief overview of the four methods with the most representative features can be found in Table 1. For comparative purpose, the liquor ratio of SF in each solvent was kept at 1:200 (*w*/*v*) for all methods. 

After each degumming process, the remaining SF of each batch was washed twice with 1.5 L of distilled water at 60 °C for 5 min to remove any unbound SS. The obtained SF was dried for 24 h in a fume hood until constant weight. The mass loss after the different degumming methods was calculated, following the method previously described expressed in the equation 1, where *m*_before degumming_ and *m*_after degumming_ are the dry mass before and after degumming, respectively [24].

(1) $$\mathrm{Mass}\;\mathrm{loss}\;\left(\%\right)=\left(\frac{m_{\mathrm{before}\;\mathrm{degumming}}-m_{\mathrm{after}\;\mathrm{degumming}}}{m_{\mathrm{before}\;\mathrm{degumming}}}\right)\times 100$$

### 2.3. Preparation of Silk Fibroin Nanoparticles

The preparation of SFNs was based on the method described by Montalbán et al. [7]. In brief, the silk fibroin fibers obtained from the different degumming methods were dissolved in [emim^+^][AcO^−^] (10 wt %) by using high-power ultrasounds. Then, to reduce the viscosity of the mixtures, 3 mL of MilliQ water (18 mΩ/cm) were added to 5 g of the mixtures SF‒[emim^+^][AcO^−^] solution and then heated to 60 °C. Subsequently, the solution was sprayed as an aerosol by a thermostatically controlled 0.7 mm two-fluid nozzle (from a Mini Spray Dryer B-290, BÜCHI Labortechnik, Flawil, Switzerland, Part No. 044698) with compressed N2 (1 bar) onto 100 mL of cold methanol (−20 °C) gently stirred. The resultant suspension was kept under stirring for 2 h and the nanoparticles were separated by centrifugation and subsequent washing steps with methanol (1×) and water (3×) and freeze dried by using an Edwards Modulyo 4K Freeze Dryer (Edwards High Vacuum International, Crawlet, UK) at −55 °C and 0.5 mbar for 72 h. Methanol and [emim^+^][AcO^−^] were recovered and purified from the supernatant by simple distillation.

### 2.4. Sodium Dodecylsulfate-Polyacrylamide Gel Electrophoresis (SDS-PAGE)

Electrophoresis was performed on the obtained SF solutions in [emim^+^][AcO^−^] according to the Laemmli protocol [46] in order to compare the protein integrity after the different degumming processes. The different samples of SF dissolved in the ionic liquid as described above, were diluted (1% *w*/*v*) with MilliQ water and mixed with loading buffer (1:1) containing β-mercaptoethanol (10% *v*/*v*) and heating the mixture at 95 °C for 5 min for protein denaturation. Next, protein concentrations were unified at 50 μg per lane of sample. The electrophoresis was performed following the protocol described by the manufacturer on an Amersham ECL Gel horizontal electrophoresis system (Code No. 28-9906-08) (GE Healthcare Europe, GmbH, Freiburg, Germany) connected to a BioRad Power supply at 100 V (BioRad, Hercules, CA, USA), by using an acrylamide gel with a 4–20 wt % gradient for better protein resolution (Amersham GE, Code No. 28-9901-59) and an Amersham ECL Gel Running buffer, 250 mL (Code No 28-9902-52). An aliquot of 5 μL of ColorBurst™ Electrophoresis Marker (Sigma-Aldrich, St. Louis, MO, USA) was loaded as reference. After electrophoresis, the gels were stained with 0.25% Coomassie Brilliant Blue (Acros Organics, Geel, Belgium), fixed, discolored in ethanol/water mixture, and photographed for analysis.

### 2.5. Morphological, Physicochemical, and Structural Characterization of SF Fibers and SFNs

For the morphological characterization of the SF and the SFN, electron microscopy images were recorded by an SEM JEOL JSM 6100 (Tokyo, Japan) and FE-SEM Thermo Scientific Apreo S (Brno, Czech Republic), respectively. For the SF samples, fibers were stuck on an aluminum stub with a thin self-adherent carbon film and later sputtered with gold. The SEM was operated at 20 kV. For SFNs, a diluted SFN dispersion was dropped on a mica disc (V1 highest grade) (Ted Pella, Inc., Redding, CA, USA), air dried, and later sputtered with platinum for 5 min resulting in a 5.13 nm film thickness (Leica, EM ACE600, Leica Microsystems Inc, Concord, ON, Canada). Mica discs were pretreated by removing the upper layers with scotch tape three times before placing the sample.

The thermal properties of SF were measured by thermal gravimetric analyzer (TA instruments, SDT 2960 simultaneous TGA-DTA, Waters LLC, New Castle, DE, USA) in a temperature range of 25–800 °C with a heating rate of 10 °C/min under an inert nitrogen atmosphere in an open bin. Weight loss and temperature difference between the sample and an inert reference were simultaneously recorded and plotted against temperature for Thermogravimetric Analysis (TGA) and Differential Thermal Analysis (DTA), respectively.

Infrared spectral data obtained by Attenuated Total Reflectance Fourier Transform Infrared Spectroscopy (ATR-FTIR) were collected in order to compare the effect of the different degumming processes in the secondary structure of the fibers. The calculation of the Crystallinity index (CI%) and the changes in the band fitting of the Amide I region (1735–1580 cm^−1^) of the spectra were chosen as the most informative for this purpose [47,48]. ATR-FTIR spectra were recorded in a Nicolet iS5 spectrometer coupled to a diamond crystal iD7 ATR module (Thermo Fischer Scientific, Waltham, MA, USA). OMNIC Software V9.9.471 (Thermo Fischer Scientific, Waltham, MA, USA) was used for controlling the equipment and the post processing of spectral data, including baseline and ATR corrections. Measurements were made with a resolution of 4 cm^−1^ in the spectral range of 4000–550 cm^−1^ by collecting 64 scans using N-B strong apodization and mertz phase correction. A background was collected before each spectrum measurement. All samples were vacuum dried prior to assessment to minimize the contribution of water to the spectra.

The CI% of the SF samples was calculated using the following equation:

(2) $$\mathrm{Crystallinity}\;\mathrm{Index}\;\left(\mathrm{CI}\%\right)=\;\frac{A_{1260}}{A_{1235}}\times 100$$

where *A*_1260_ and *A*_1235_ represent the measured absorbance at 1260 and 1235 cm^−1^, respectively, after performing a baseline correction (See an example at Figure S1, from the electronic supplementary information).

Complementarily, the secondary structure of the SF samples was also assigned by band fitting of the Amide I region, (1600–1700 cm^−1^) of the spectra by using OMNIC V9.9.471 software following the approach described by Hu et al. [49]. Finally, the resulting Gaussian bands were area-normalized, and the relative area of the individual bands was assigned to the secondary structure of the protein according to the position of the peak, as can be observed in Table S1 of the electronic supplementary information. The aforementioned calculation was made assuming that all the C=O stretching vibrations have the same excitation coefficient, which means that their areas are proportional to the fraction of each secondary structural component [50]. An example of the band assignation can be found in the Figure S2 of the electronic supplementary information.

The characterization of the nanoparticles regarding their size and surface characteristics was performed by dynamic light scattering by using a Malvern Zetasizer Nano ZSP (Malvern Intruments Ltd, Worcestershire, UK), equipped with a laser of 4 mW power and 633 nm wavelength. Intensity weighted mean hydrodynamic diameter, expressed as Z-Average, and Z-potential were measured by DLS and phase analysis light scattering (PALS) techniques, respectively. Samples were seen not to absorb at 633 nm in measurement conditions. A 1 mg/mL SFNs suspension was prepared by ultrasonication for 1 min at 30% amplitude with pulses of 15 s before loading into the disposable DTS1070 capillary cell for Z-potential (Malvern Intruments Ltd, Worcestershire, UK). The backscattered light was measured at 173° relative to the source after 120 s of equilibration time at 25 °C. The integrated Malvern software calculates the Z-Average and size distribution from fitting the autocorrelation function by CUMULANT analysis and multiple exponential decays by non-negative least square (general purpose under Malvern software options), respectively. Size results shown are the average of 3 measurements, were each measurement consisted of 12 runs of 10 s with no delay between measurements. The Z-potential was calculated through Henry′s equation using electrophoretic mobility of SFNs. Smoluchoski approximation (κα = 1.5) was assumed. Z-potential results are the average of 6 measurements taken in a fully automated way by the software with a minimum of 12 runs.

The surface charge density (σ) of a spherical colloidal particle can be obtained following the method proposed by Makino and Ohshima on the basis of the Z-potential and the ionic strength of the medium [51].

Detailed equations and parameter values used for the calculation of the surface density charge are described in supplementary information (See Table S2).

### 2.6. Statistical Analysis

Data were presented as mean ± SD (standard deviation), calculated from three independent samples per condition by using Graphpad Prism 8.0.1 software (GraphPad Software, San Diego, CA, USA). As normality (Kolmogorov-Smirnov, *p* > 0.05) and homoscedasticity (Levene, *p* > 0.05) were met, the statistical significance was determined using the parametric tests of Tukey (*p* < 0.05) and ANOVA (*p* < 0.05) for the comparisons of two or more groups, respectively.

## 3. Results and Discussion

### 3.1. Degumming Results

The raw SC and the SF obtained after the degumming process, were weighed and their mass differences are presented in Table S3 (supplementary information). From the results, the weight loss follows the series D4 < D1 < D2 < D3. The D1 and D2 methods achieved similar weight loss of 31 and 32.4%, respectively. The D3 method represents a remarkable increase in the severity of the treatment in terms of time with respect to D2, and the weight loss reached 44.4%, the highest of the different tested methods. D4 method yielded the minor loss of mass, with only 25.9%. Assuming that only 25–30% of the total weight in the cocoons corresponds to the SS fraction [13,52,53], we can infer that not only sericin but also fibroin have been lost in the D3 treatment, due of the harsh conditions for the fibers, a phenomenon previously described [24].

**Figure 1:**
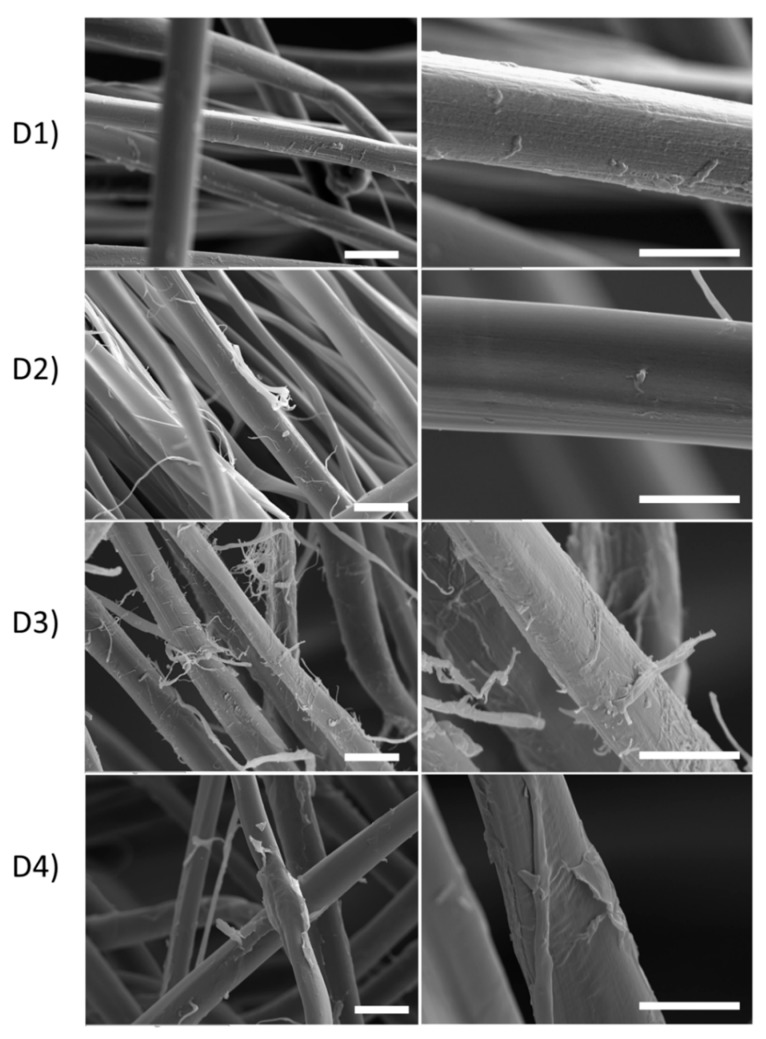
Comparative images obtained with scanning electron microscopy of the degummed SF from the following degumming process at two magnifications: (**D1**) Autoclave, (**D2**) Na_2_CO_3_ 30′, (**D3**) Na_2_CO_3_ 120′ and (**D4**) Ultrasound. (Scale bar: 20 μm).

The integrity of the SF fibers after the different degumming treatments were studied by scanning electron microscopy. As can be seen in Figure 1, there are notable differences in the surface of the SF fibers, due to the relative damage suffered during each treatment.

The images of the SF samples at different magnifications presented visible differences in the surface of the fibers. It can be observed that degummed SF-D1 showed a mostly smooth surface, but small remains of SS are still attached to the SF fibers. The SF-D2 presented the smoothest surface, almost completely free of SS. The degummed SF-D3 revealed a highly damaged structure, with no clear signs of SS but fully coated with detached microfibers. These irregularities reflect the intense degradation produced by this degumming method. The SF-D4 did not present unraveled microfibers, but the irregular surface revealed the presence of SS, as reported previously [54,55].

### 3.2. SF Secondary Structure Analysis

CI(%) determination by ATR-FTIR spectroscopy is a useful tool for assessing the effect of the degumming process due to the remarkable differences of the SS and SF secondary structures [56], which are translated to the different signals in the amide III band [57,58]. The CI values were expected to increase as SS is removed due to the fact that SF presents a more ordered structure than SS [59,60]. The CI% was calculated with the ratio of the absorption bands at 1260 cm^−1^ (Crystallinity, β-Sheet) [61] and 1235 cm^−1^ (amorphous, random coil) [62] bands from the recorded spectra (See Figure S3). Results for the CI% of the different samples are shown in Table S3. All SF samples showed an increase in CI with respect to the raw silk cocoon, as expected. Not surprisingly, SF-D1 and SF-D3 presented the highest CI, both 59%, but, in our opinion, due to different factors. On the one hand, the alkaline carbonate treatment has the potential to partially degrade the SF fibers, especially those regions with lower crystallinity, resulting in an increase in the overall CI%, an effect previously reported in literature [47]. This result is in accordance with the highest mass loss among samples (44.4%) observed during the treatment. On the other hand, the autoclave process with high pressure and temperature would induce an increase in the overall -Sheet content through a process of internal rearrangement of the chains facilitated by water molecules also described by Xu et al. [63]. The D2 method had a moderate increase in terms of CI% (56%) compared to D1, with a similar mass loss. The ultrasound-degummed SF showed the lowest CI% (52%), which correlates well with the fact that it is the sample with the lowest mass loss (25.9%), and SS are probably still adhered to the surface of the SF, lowering the overall CI%.

**Figure 2:**
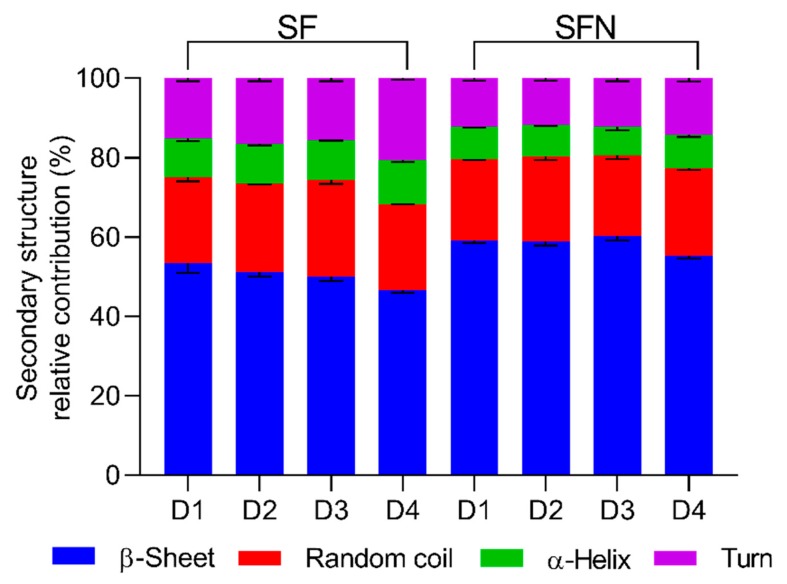
Secondary structure relative contribution of the silk cocoons and silk fibroins in the different stages of the process, obtained by band fitting of the Amide I infrared absorption band. SF—Degummed silk fibroin, and SFN—Silk fibroin nanoparticles prepared from SF degummed by: (**D1**) Autoclave, (**D2**) Na_2_CO_3_ 30′, (**D3**) Na_2_CO_3_ 120′ and (**D4**) Ultrasound. *(Please refer to the online version for the color representation of the figure).*

Comparative views of the spectra of degummed silk fibroin and the lyophilized nanoparticles can be viewed in the Figure S3 of the supplementary information. In order to investigate the effect of the degumming process on SFN formation, the Amide I infrared absorption band was chosen as a marker of the secondary structure of SF, following the procedure described in Section 2.5. The secondary structure features of the degummed SF are summarized in Figure 2 and Table S4, along with a statistical comparison (two-way ANOVA), in the Table S5 of the supplementary material.

After the degumming process of the SC, the random and α-helix structures were largely reduced while the β-Sheet content (%) increased in all SF samples due to the removal of the SS. Resultant values agree with the previously proposed for SF [49,64,65]. SF-D1 showed the highest β-Sheet content. As was stated before, the severe conditions of pressure and temperature would induce a certain internal rearrangement of the chains, promoted by water molecules, resulting in an increase of β-Sheet [63]. No statistical differences could be seen in β-Sheet content between SF-D2 and SF-D3, while SF-D4 presented the lowest amount of β-sheet. SF-D2 and SF-D3 samples presented the highest relative percentage of random coil structure with 22.4 and 24.5%, respectively.

When processing the SF into SFN, the overall β-Sheet structure increased while the rest of secondary structures were reduced for all samples, in agreement with similar works [45]. It could be hypothesized that the relative increase in the β-Sheet content may be caused by degradation of the amorphous regions in peptides of low molecular weight. Therefore, the small peptides of the protein could be lost during the SF coagulation and subsequent washing steps.

Interestingly, the β-Turn structures, which are short hydrophilic chains that connect the β-Sheet strands, showed the highest reduction among all secondary structures. This finding suggests that during the dissolution of the SF in ionic liquid and later nanoprecipitation in methanol, these β-Turns peptides were partially lost. SFN-D1, SFN-D2, and SFN-D3 samples showed similar relative amounts of secondary structure. However, SFN-D4 showed a higher content of β-turn and random coil.

### 3.3. Sodium Dodecylsulfate-Polyacrylamide Gel Electrophoresis (SDS-PAGE)

**Figure 3:**
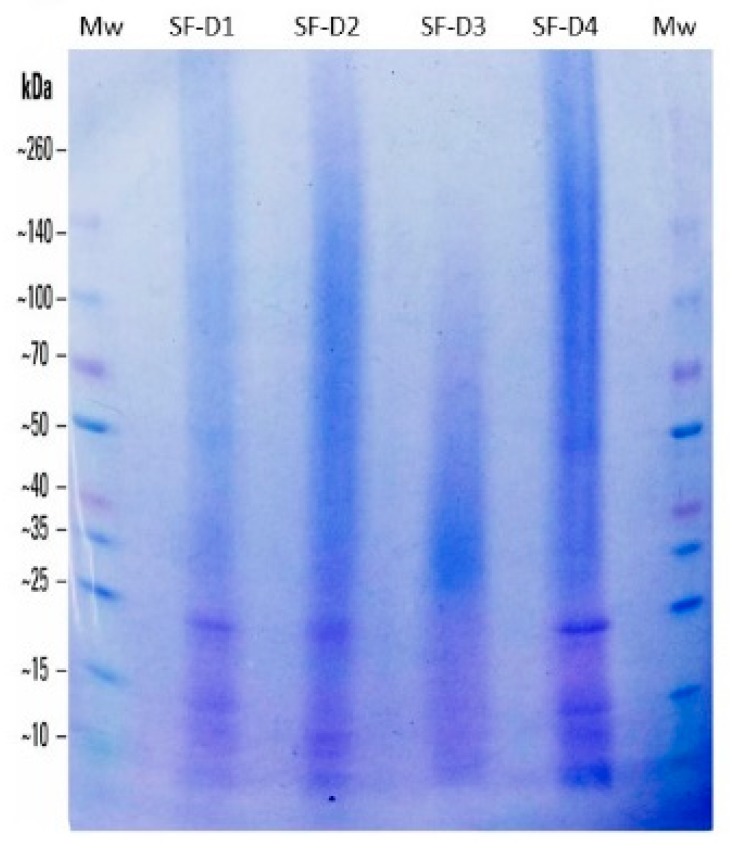
Dodecyl Sulfate-Polyacrylamide Gel Electrophoresis (SDS-PAGE) Analysis of the protein components of SF after the different degumming process, namely: (**D1**) Autoclave; (**D2**) Short alkaline boiling; (**D3**) Intensive alkaline boiling and (**D4**) Ultrasonication probe. Lane *M*_W_: Spectra Multicolor Protein Ladder 10–260 kDa (Thermo), (4–20% gradient Gel Amersham GE-HC). *(Please refer to the online version for the color representation of the figure).*

The effect of the degumming method on the molecular weight (*M_w_*) of the SF chains was studied by gel electrophoresis, and the result is shown in Figure 3. Native SF consists of a heavy chain (H), a light chain (L), and a glycoprotein (P25), and their respective molecular weights are 391, 26, and 24 kDa (30 kDa including the *N*-linked oligosaccharides). The H and L-chains are bonded together by a disulfide bridge [66] and complexed with P25 in a 6:6:1 molar ratio [67]. This protein, P25, was only slightly visible in the electrophoresis gel (Figure 3) in the lane of SF-D4.

The studied SF samples presented large differences between them, as seen by electrophoresis. SF-D1 showed a light smear along the whole lane, and clear bands at 26 and 15 kDa can be observed. SF-D2 appeared as a smear of peptide sizes from 140 kDa downwards; the L-chain of fibroin is clearly visible at 26 kDa. SF-D3 presented a size distribution as a smear between 100 and 10 kDa with a darker zone in the 30–40 kDa, and no bands were detected at 26 or 24 kDa. SF-D4 showed a heterogeneous smear along the whole line with darker zones at 140, 100, and 50 kDa and clear bands at 26 and 15 kDa.

The smears in the upper-middle molecular weight distribution can be interpreted as fragments of the H-chain as a result of the degradation from the degumming process [26]. The SF-D1 was seen to be the least aggressive treatment for SF, which is denoted by the light smear with only a small fraction of H-chain degraded, while the major fraction of the protein could not enter the gel due to the size of the chains. On the other hand, the L-chain of 26 kDa is clearly evident, which accentuates the hypothesis of the SF-D1 treatment producing lower SF degradation. Similar results for autoclave degumming have been published [21]. SF-D2 and SF-D4 presented a moderately dark smear in the top portion of the gel (ca. 260–50 kDa) similar to that previously described [6], with bands at 120 kDa (SS) [68], 50 kDa (H-chain of SF), 35 kDa (H-chain of SF), 26 kDa (L-chain of SF), and 15 kDa (SS) [13]. The collections of bands and the smear at the top portion of the gel confirmed the SF H-chain degradation and remaining SS, as seen in the optical and electronic microscope images. This was especially evident for SF-D4, reflecting the SEM images (Figure 1) and the low CI%. SS comprises a big family of proteins, and they can be classified into three groups according to their position in the fiber, the outer, the middle and the inner layers. The inner layers contain the smallest and the least water-soluble proteins. These inner layers of SS have a molecular weight of 14.4 kDa [13]. The bands at 15 kDa seen in SF-D1, SF-D2 and SF-D4 could correspond to these inner SS proteins. This fact correlates with the optical microscopy images, where SF-D1 and SF-D2 showed the remains of small still attached SS and SF-D4 presented bands of SS with heavier molecular weights. Among the treatments studied, alkaline boiling for 120 min produced the largest degradation of the H and L-chains, as no signs of high molecular weight fragments (>100 kDa) or light chain band at 26 kDa could be identified. This treatment clearly narrows the distribution of molecular weight and concentrate them in the bottom portion of the distribution range.

Through an analysis of the gel, we conclude that the different degumming treatments produced different degrees of degradation of the SF chains: D1 < D4 < D2 < D3. Thus, the alkaline boiling for 120 min appeared to be the most aggressive process for SF degumming, which agrees with other authors [16,32].

### 3.4. Thermal Properties

**Figure 4:**
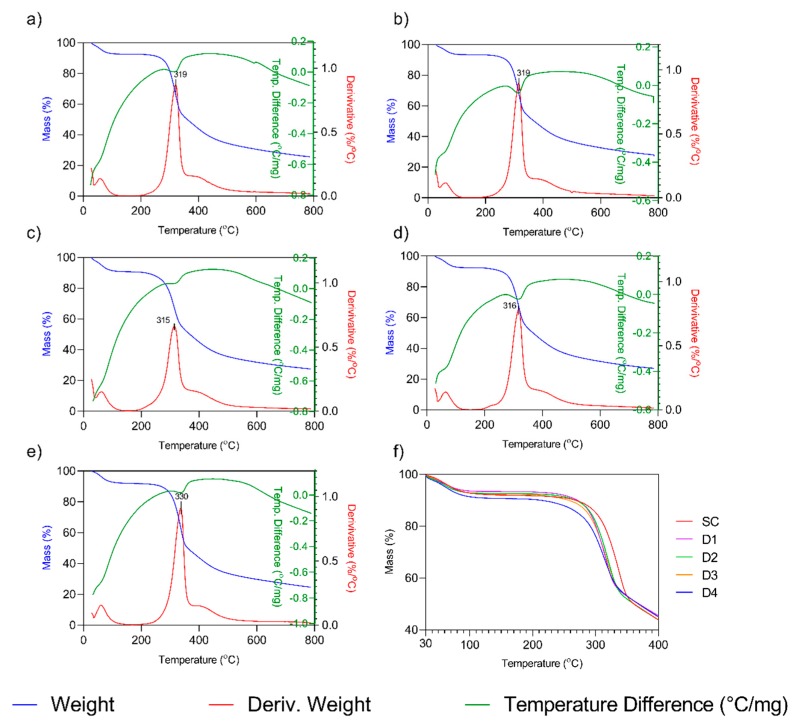
Thermogravimetric and Differential Thermal Analysis (TGA-DTA) of the SC and SF after the different degumming process: (**a**) D1, Autoclave; (**b**) D2, Na_2_CO_3_ 30′; (**c**) D3, Na_2_CO_3_ 120′; (**d**) D4, Ultrasounds; (**e**) SC, Silk cocoon, and (**f**) TGA curves of all samples. *(Please refer to the online version for the color representation of the figure.).*

The obtained results for TGA-DTA of the SC and the SF fibers after the four different degumming processes are depicted in Figure 4, along with thermal the decomposition rate temperature (*T_dm_*) denoted by the peak of the first derivate. *T_dm_* corresponds to an endothermic transition (negative peak of DTA curve). The mass loss in the first transition at T < 100 °C, were due to the evaporation of water content. As can be seen, in all cases, the weight residue percentage is reduced sharply after 315–319 °C due to the degradation of SF, which is attributed to thermal degradation of β-Sheet ordered structures [69]. Both mild degumming methods for SF (D1 and D2) showed equal *T_dm_* of 319 °C. Fibers degummed by the more aggressive treatments, D3 and D4 processes, showed the lowest *T_dm_* at 315 and 316 °C, respectively. Comparatively, the cocoon sample showed a significantly higher *T_dm_* reaching 330 °C (Figure 4e). This higher thermal stability of the cocoon has been previously described [2].

### 3.5. Size and Morphology of the SFNs

**Figure 5:**
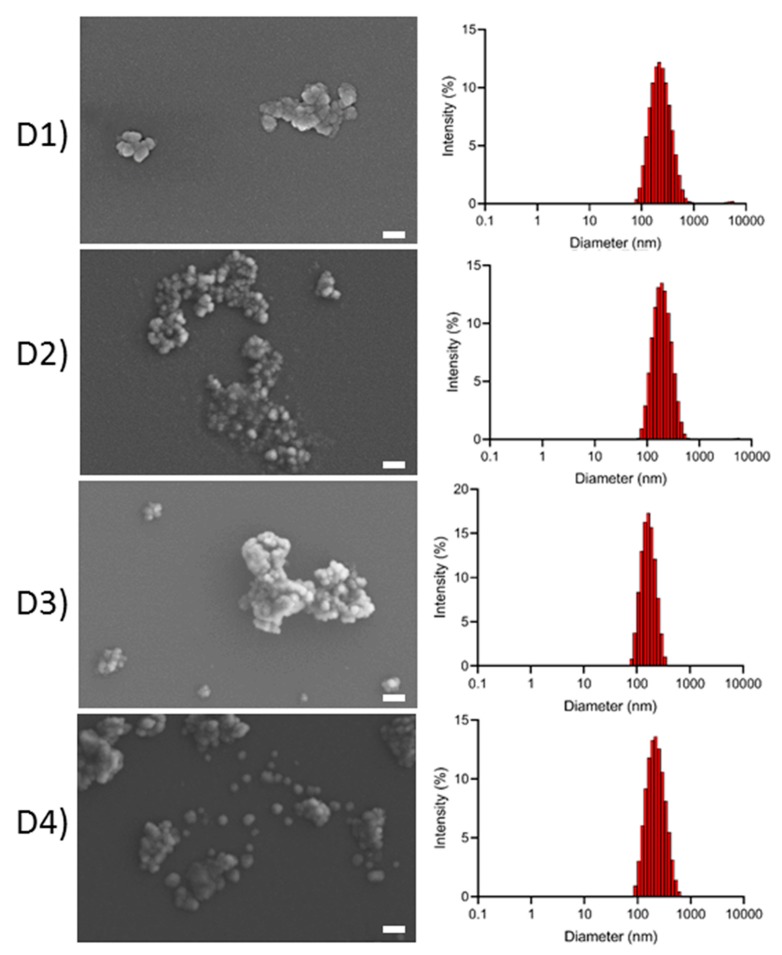
Field Emission Scanning Electron Microscopy (FE-SEM) images of SFN with the corresponding size distributions by intensity measured in Dynamic Light Scattering (DLS) prepared from SF degummed by: (**D1**) Autoclave, (**D2**) Na_2_CO_3_ 30′, (**D3**) Na_2_CO_3_ 120′ and (**D4**) Ultrasounds. Scale bar = 200 nm.

The four degummed SFs were processed to produce SFNs as stated in the experimental section, and their hydrodynamic features were determined by DLS to assess how the degumming process affects the final size distribution, polydispersity index (PdI), Z-potential, and surface charge density of nanoparticles. The size distribution and FE-SEM images of the SFN are shown in Figure 5, and the values of Z-average (d.nm) and PdI, calculated with the cumulant approach, are presented in Table 2. All SFNs presented a monomodal size distribution with low values of PdI (<0.2). FE-SEM images of the SFN are also shown in Figure 5. In the dried state, the SFNs appeared as nanoparticle clusters. However, FE-SEM images revealed the almost spherical morphology of the nanoparticles.

Nonetheless, there were significant differences in both Z-Average and size distributions among the SFN samples (*p* < 0.05). As depicted in Table 2, both the Z-average and PdI of the nanoparticles varied in the order: D1 > D4 > D2 > D3. D3-SFN presented the narrowest size distribution and the smallest Z-average of all samples, and this correlates with the highest degradation of SF peptides observed in the gel analysis. Thus, the length of the peptide chains would affect the final size of the nanoparticles. The smaller the size of the peptide chains, the smaller the resultant nanoparticles.

All samples showed a negative Z-potential ranging from −24.7 to −30.2 mV (Table 2) that support the stability of the aqueous suspensions of nanoparticles due to electrostatic repulsion forces among particles negatively charged. The nanoparticles obtained from both alkaline degummed SF showed similar Z-potentials, but higher in absolute value than the “water only” treatments. This fact can be explained by the higher pH value of the solvent in the treatment, which could produce a highest number of deprotonated carboxyl groups, conferring a more negative net charge to the SFNs.

The surface charge density can be more descriptive of the effect of the degumming treatment on the characteristics of the nanoparticles because not only the Z-potential but also the size of the nanoparticles are variables in the calculation of the surface charge density. As can be seen on Table 2, the surface charge density reaches values of about 2.5 × 10^−3^ C/m^2^. The nanoparticles obtained from degummed fibroins in water-only treatments presented a lower density around 2.0 × 10^−3^ C/m^2^. Considering the nanoparticles as spheres with a diameter equivalent to the Z-average, the concentration of negative charges per unit of mass can be calculated, which ranged from 7.31 × 10^−1^ µmol/g for SFN-D3 to 4.32 × 10^−1^ for SFN-D4. Differences in the number of solvent accessible carboxylate groups have remarkable effect on the functionalization of the surface by using carbodiimide coupling chemistry.

## 4. Conclusions

The main aim of this work was to study the effects of four degumming methods, namely autoclaving, short, and intensive alkaline boiling (30 and 120 min, respectively) and high-power ultrasounds on SF integrity and, consequently, on the formed SFN. It was hypothesized that with a higher degradation, smaller nanoparticles could be achieved, due to the smaller polymer chains used as building blocks.

The integrity of SF was analyzed by gel electrophoresis, whereby smaller molecular weight distributions were interpreted as reflecting a higher degree of degradation. The autoclave method was the least aggressive as regards SF integrity, while yielding good SS removal. Alkaline methods were seen to be more aggressive with the SF compared to the rest of the treatments. An increase in alkali processing time, increased fibroin degradation, further reducing the molecular weight distribution, as expected. While small quantities of SS were detected when SF fibers were treated with the mild process, no remaining SS was observed after the long process. The ultrasound method underperformed in terms of SS removal in the experimental conditions of this work but produced comparatively lower damage to the SF fibers. An overall increase in β-Sheet structure was observed during SFN preparation, with the consequent reduction of less ordered structures (α-helix or random coil).

There was a positive correlation between SF degradation and a reduction in the mean size and size distribution of SFN. However, the significant changes in Z-potential and surface charges density between SFNs coming from different treatments can be attributed to the nature of the degumming solvents (i.e. alkaline solution or ultrapure water).

Although the degumming using sodium carbonate solution for 30 min has been stated among the studied alternatives as the best degumming process for the production of silk fibroin implantable biomaterials [24], allowing to retain SF integrity and achieve complete sericin removal, for the preparation of nanodrug delivery systems, the harsh treatment D3 is the most effective degumming process, not only because of the size and Z-potential, but also because of the increasing of carboxylate groups in SFN-D3, which improves availability of reactive sites on the particle surface. These carboxylate groups can be easily functionalized by means of carbodiimide coupling [70], providing a more favorable surface as nano carrier-based drug delivery system.

As a final remark, the degumming method implemented for silk fibroin purification must be taken into account as a key step in the protocol used for SFN preparation, as the integrity of the protein affects to the mean size, the size distribution, and the surface charge density of the particles.

## Figures and Tables

**Table 1:** Overview of the used degumming methods.

Reference	Process	Solvent	T (°C)	Time (min)
D1	Autoclave	MilliQ water	121	30
D2	Short Alkaline Boiling	Na_2_CO_3_ 0.02 mol/L	100	30
D3	Intensive Alkaline Boiling	Na_2_CO_3_ 0.02 mol/L	100	120
D4	Ultrasonication with probe *	MilliQ water	60	60

* Branson Sonifier SFX-550 (Emmerson Ultrasonic Corporation. Dansbury, CT, USA) equipped with a disruptor horn and a 1/8” diameter tapered microtip. Sonication was set for 30 min with 50% amplitude, and the maximum temperature was set at 60 °C.

**Table 2:** Hydrodynamic diameter, Polydispersity Index, and Z-potential of SFN obtained by using SF from the following degumming process: (**D1**) Autoclave, (**D2**) Na_2_CO_3_ 30′, (**D3**) Na_2_CO_3_ 120′ and (**D4**) Ultrasounds.

Sample	Z-Average (nm) ^a^	PdI	Z-Potential (mV) ^a^	Surface Charge Density (C/m^2^)	Negative Charges (mM/g)
**SFN-D1**	214 ± 4	0.185 ± 0.003	−26.4 ± 0.5	−2.16 ± 0.04 × 10^−^^3^	4.48 × 10^−1^
**SFN-D2**	179 ± 1	0.146 ± 0.008	−30.2 ± 1.6	−2.53 ± 0.12 × 10^−3^	6.29 × 10^−1^
**SFN-D3**	156 ± 3	0.087 ± 0.002	−30.2 ± 1.8	−2.57 ± 0.06 × 10^−3^	7.31 × 10^−1^
**SFN-D4**	207 ± 4	0.152 ± 0.004	−24.7 ± 1.6	−2.01 ± 0.06 × 10^−3^	4.32 × 10^−1^

^a^ Results are shown as mean ± standard deviation, *n* = 3.

## Supplementary Materials

The following are available online at https://www.mdpi.com/2073-4360/11/12/2045/s1, Figure S1. Example of base line traced in Amide III absorption band for the crystallinity index calculation.; Table S1. Assignment of the vibration bands in Amide I.; Figure S2. FSD of Amide I absorption band and example of band fitting.; Table S2: List of parameters used to calculate the surface charge density.; Table S3. Degumming efficiency expressed in terms of mass loss and Crystallinity index (CI) of SF samples: D1) Autoclave, D2) Na2CO3 30′, D3) Na2CO3 120′, and D4) Ultrasounds.; Figure S3. ATR-FTIR spectra of silk fibroins (SF) and silk fibroin nanoparticles (SFN) degummed by: D1) Autoclave, D2) Na2CO3 30′, D3) Na2CO3 120′ and D4) Ultrasound Amide III absorption band for the crystallinity index calculation. Insets with magnification of the Amide I region of the spectra, used for determination of the secondary structures are included. (Please refer the online version for the color representation of the figure); Table S4: Relative contribution of secondary structure features of the Amide I in the different stages of the process. SC) Silk Cocoons, internal or external faces, SF) Degummed silk fibroin and SFN) Silk fibroin nanoparticles prepared from SF degummed by: D1) Autoclave, D2) Na_2_CO_3_ 30′, D3) Na_2_CO_3_ 120′ and D4) Ultrasound obtained by FSD analysis of the Amide I infrared absorption band. *n* = 3, average ± standard deviation.; Figure Table S5: Analysis of variance (ANOVA) of the relative contribution of secondary structure (*n* = 3).

## References

[1] Holland C., Numata K., Rnjak-Kovacina J., Seib F.P. (2019). The Biomedical Use of Silk: Past, Present, Future. Adv. Healthc. Mater..

[2] Mazzi S., Zulker E., Buchicchio J., Anderson B., Hu X. (2014). Comparative thermal analysis of Eri, Mori, Muga, and Tussar silk cocoons and fibroin fibers. J. Therm. Anal. Calorim..

[3] Rockwood D.N., Preda R.C., Yücel T., Wang X., Lovett M.L., Kaplan D.L. (2011). Materials fabrication from *Bombyx mori* silk fibroin. Nat. Protoc..

[4] Ajisawa A. (1998). Dissolution of silk fibroin with calciumchloride/ethanol aqueous solution. J. Seric. Sci. Jpn..

[5] Phillips D.M., Drummy L.F., Conrady D.G., Fox D.M., Naik R.R., Stone M.O., Trulove P.C., De Long H.C., Mantz R.A. (2004). Dissolution and Regeneration of *Bombyx mori* Silk Fibroin Using Ionic Liquids. J. Am. Chem. Soc..

[6] Lozano-Pérez A.A., Montalbán M.G., Aznar-Cervantes S.D., Cragnolini F., Cenis J.L., Víllora G. (2015). Production of silk fibroin nanoparticles using ionic liquids and high-power ultrasounds. J. Appl. Polym. Sci..

[7] Montalbán M., Coburn J., Lozano-Pérez A., Cenis J., Víllora G., Kaplan D. (2018). Production of Curcumin-Loaded Silk Fibroin Nanoparticles for Cancer Therapy. Nanomaterials.

[8] Hernández-Fernández F.J., de los Ríos A.P., Tomás-Alonso F., Gómez D., Rubio M., Víllora G. (2007). Integrated reaction/separation processes for the kinetic resolution of rac-1-phenylethanol using supported liquid membranes based on ionic liquids. Chem. Eng. Process. Process Intensif..

[9] Hernández-Fernández F.J., de los Ríos A.P., Tomás-Alonso F., Gómez D., Víllora G. (2008). On the development of an integrated membrane process with ionic liquids for the kinetic resolution of rac-2-pentanol. J. Memb. Sci..

[10] Rogers R.D., Seddon K.R. (2003). Ionic Liquids—Solvents of the Future?. Science.

[11] Wang H., Zhang Y., Shao H., Hu X. (2005). A study on the flow stability of regenerated silk fibroin aqueous solution. Int. J. Biol. Macromol..

[12] Aznar-Cervantes S.D., Lozano-Pérez A.A., García Montalbán M., Víllora G., Vicente-Cervantes D., Cenis J.L. (2015). Importance of refrigeration time in the electrospinning of silk fibroin aqueous solutions. J. Mater. Sci..

[13] Wang Y.-J., Zhang Y.-Q. (2011). Three-layered sericins around the silk fibroin fiber from *Bombyx mori* cocoon and their amino acid composition. Adv. Mater. Res..

[14] Freddi G., Mossotti R., Innocenti R. (2003). Degumming of silk fabric with several proteases. J. Biotechnol..

[15] Mahmoodi N.M., Moghimi F., Arami M., Mazaheri F. (2010). Silk degumming using microwave irradiation as an environmentally friendly surface modification method. Fibers Polym..

[16] Wang R., Zhu Y., Shi Z., Jiang W., Liu X., Ni Q.Q. (2018). Degumming of raw silk via steam treatment. J. Clean. Prod..

[17] Cao T.T., Zhang Y.Q. (2016). Processing and characterization of silk sericin from *Bombyx mori* and its application in biomaterials and biomedicines. Mater. Sci. Eng. C.

[18] Pérez-Rigueiro J., Elices M., Llorca J., Viney C. (2002). Effect of degumming on the tensile properties of silkworm (*Bombyx mori*) silk fiber. Appl. Polym. Sci..

[19] Yuksek M., Kocak D., Beyit A., Merdan N. (2012). Effect of Degumming Performed with Different Type Natural Soaps and Through ultrasonic method on the properties of silk fiber. Adv. Environ. Biol..

[20] Wang F., Zhang Y.Q. (2017). Effects of alkyl polyglycoside (APG) on *Bombyx mori* silk degumming and the mechanical properties of silk fibroin fibre. Mater. Sci. Eng. C.

[21] Kim H.J., Kim M.K., Lee K.H., Nho S.K., Han M.S., Um I.C. (2017). Effect of degumming methods on structural characteristics and properties of regenerated silk. Int. J. Biol. Macromol..

[22] Kumar S., Singh S.K. (2017). Fabrication and characterization of fibroin solution and nanoparticle from silk fibers of *Bombyx mori*. Part. Sci. Technol..

[23] Wang Y., Guo J., Zhou L., Ye C., Omenetto F.G., Kaplan D.L., Ling S. (2018). Design, Fabrication, and Function of Silk-Based Nanomaterials. Adv. Funct. Mater..

[24] Nultsch K., Bast L.K., Näf M., El Yakhlifi S., Bruns N., Germershaus O. (2018). Effects of Silk Degumming Process on Physicochemical, Tensile, and Optical Properties of Regenerated Silk Fibroin. Macromol. Mater. Eng..

[25] Wang L., Luo Z., Zhang Q., Guan Y., Cai J., You R., Li X. (2019). Effect of Degumming Methods on the Degradation Behavior of Silk Fibroin Biomaterials. Fibers Polym..

[26] Genç G., Narin G., Bayraktar O. (2009). Spray drying as a method of producing silk sericin powders. J. Arch. Mater. Manuf. Eng..

[27] Lalit Jajpura A.R. (2015). The Biopolymer Sericin: Extraction and Applications. J. Text. Sci. Eng..

[28] Nultsch K., Germershaus O. (2017). Silk fibroin degumming affects scaffold structure and release of macromolecular drugs. Eur. J. Pharm. Sci..

[29] Gulrajani M.L., Gupta S.V., Gupta A., Suri M. (1996). Degumming of silk with different protease enzymes. Indian J. Fibre Text. Res..

[30] Freddi G., Allera G., Candiani G. (2008). Degumming of silk fabrics with tartaric acid. J. Soc. Dye. Colour..

[31] Khan M.M.R., Tsukada M., Gotoh Y., Morikawa H., Freddi G., Shiozaki H. (2010). Physical properties and dyeability of silk fibers degummed with citric acid. Bioresour. Technol..

[32] Wang H.Y., Zhang Y.Q. (2013). Effect of regeneration of liquid silk fibroin on its structure and characterization. Soft Matter.

[33] Wang F., Cao T.T., Zhang Y.Q. (2015). Effect of silk protein surfactant on silk degumming and its properties. Mater. Sci. Eng. C.

[34] Wang Z., Yang H., Li W., Li C. (2019). Effect of silk degumming on the structure and properties of silk fibroin. J. Text. Inst..

[35] Allardyce B.J., Rajkhowa R., Dilley R.J., Atlas M., Kaur J., Wang X. (2016). The impact of degumming conditions on the properties of silk films for biomedical applications. Text. Res. J..

[36] Lee J.H., Song D.W., Park Y.H., Um I.C. (2016). Effect of residual sericin on the structural characteristics and properties of regenerated silk films. Int. J. Biol. Macromol..

[37] Park B.K., Um I.C. (2018). Effect of molecular weight on electro-spinning performance of regenerated silk. Int. J. Biol. Macromol..

[38] Aznar-Cervantes S.D., Vicente-Cervantes D., Meseguer-Olmo L., Cenis J.L., Lozano-Pérez A.A. (2013). Influence of the protocol used for fibroin extraction on the mechanical properties and fiber sizes of electrospun silk mats. Mater. Sci. Eng. C.

[39] Seib F.P., Jones G.T., Rnjak-Kovacina J., Lin Y., Kaplan D.L. (2013). pH-Dependent Anticancer Drug Release from Silk Nanoparticles. Adv. Healthc. Mater..

[40] Zhao Z., Li Y., Xie M.-B. (2015). Silk Fibroin-Based Nanoparticles for Drug Delivery. Int. J. Mol. Sci..

[41] Liu Q., Liu H., Fan Y. (2017). Preparation of silk fibroin carriers for controlled release. Microsc. Res. Tech..

[42] Mottaghitalab F., Farokhi M., Shokrgozar M.A., Atyabi F., Hosseinkhani H. (2015). Silk fibroin nanoparticle as a novel drug delivery system. J. Control. Release.

[43] Montalbán M.G., Carissimi G., Lozano-Pérez A.A., Cenis J.L., Coburn J.M., Kaplan D.L., Víllora G. (2018). Biopolymeric Nanoparticle Synthesis in Ionic Liquids. Recent Advances in Ionic Liquids.

[44] Philipp Seib F. (2017). Silk nanoparticles—An emerging anticancer nanomedicine. AIMS Bioeng..

[45] Aznar-Cervantes S.D., Pagan A., Monteagudo Santesteban B., Cenis J.L. (2019). Effect of different cocoon stifling methods on the properties of silk fibroin biomaterials. Sci. Rep..

[46] Laemmli U.K. (1970). Cleavage of Structural Proteins during the Assembly of the Head of Bacteriophage T4. Nature.

[47] Bhat N.V., Nadiger G.S. (1980). Crystallinity in silk fibers: Partial acid hydrolysis and related studies. J. Appl. Polym. Sci..

[48] Nadiger G.S., Bhat N.V. (1985). Effect of plasma treatment on the structure and allied textile properties of mulberry silk. J. Appl. Polym. Sci..

[49] Hu X., Kaplan D., Cebe P. (2006). Determining beta-sheet crystallinity in fibrous proteins by thermal analysis and infrared spectroscopy. Macromolecules.

[50] Barth A., Zscherp C. (2002). What vibrations tell us about proteins. Q. Rev. Biophys..

[51] Makino K., Ohshima H. (2010). Electrophoretic mobility of a colloidal particle with constant surface charge density. Langmuir.

[52] Zhang Y.Q. (2002). Applications of natural silk protein sericin in biomaterials. Biotechnol. Adv..

[53] Mondal M., Trivedy K., Kumar S.N. (2007). The silk protein, sericin and fibroin in silkworm, *Bombyx mori* Linn—A review. Casp. J. Environ. Sci..

[54] Lotz B., Colonna Cesari F. (1979). The chemical structure and the crystalline structures of *bombyx mori* silk fibroin. Biochimie.

[55] Ling S., Qi Z., Knight D.P., Shao Z., Chen X. (2011). Synchrotron FTIR microspectroscopy of single natural silk fibers. Biomacromolecules.

[56] Koperska M.A., Pawcenis D., Bagniuk J., Zaitz M.M., Missori M., Łojewski T., Łojewska J. (2014). Degradation markers of fibroin in silk through infrared spectroscopy. Polym. Degrad. Stab..

[57] Marsh R.E., Corey R.B., Pauling L. (1955). An Investigation of the structure of solk fibroin. Biochim. Biophys. Acta.

[58] Altman G.H., Diaz F., Jakuba C., Calabro T., Horan R.L., Chen J., Lu H., Richmond J., Kaplan D.L. (2003). Silk-based biomaterials. Biomaterials.

[59] Shao J., Zheng J., Liu J., Carr C.M. (2005). Fourier transform Raman and Fourier transform infrared spectroscopy studies of silk fibroin. Appl. Polym. Sci..

[60] Garside P., Wyeth P. (2007). Crystallinity and degradation of silk: Correlations between analytical signatures and physical condition on ageing. Appl. Phys. A.

[61] Hu X., Kaplan D., Cebe P. (2008). Dynamic Protein—Water Relationships during Beta Sheet Formation. Macromolecules.

[62] Yang Y., Chen J., Bonani W., Chen B., Eccheli S., Maniglio D., Migliaresi C., Motta A. (2018). Sodium oleate induced rapid gelation of silk fibroin. J. Biomater. Sci. Polym. Ed..

[63] Wongpinyochit T., Johnston B.F., Seib F.P. (2018). Degradation Behavior of Silk Nanoparticles—Enzyme Responsiveness. ACS Biomater. Sci. Eng..

[64] Nakpathom M., Somboon B., Narumol N. (2009). Exploring natural silk protein sericin for regenerative medicine: An injectable, photoluminescent, cell-adhesive 3D hydrogel. J. Microsc. Soc. Thail..

[65] Bawazeer T.M., Alsoufi M.S. (2017). Surface Characterization and Properties of Raw and Degummed (*Bombyx mori*) Silk Fibroin Fiber toward High Performance Applications of “*Kisswa Al-Kabba*”. Int. J. Curr. Res..

[66] Zhou C.Z., Confalonieri F., Jacquet M., Perasso R., Li Z.G., Janin J. (2001). Silk fibroin: Structural implications of a remarkable amino acid sequence. Proteins Struct. Funct. Genet..

[67] Inoue S., Tanaka K., Arisaka F., Kimura S., Ohtomo K., Mizuno S. (2000). Silk fibroin of *Bombyx mori* is secreted, assembling a high molecular mass elementary unit consisting of H-chain, L-chain, and P25, with a 6:6:1 molar ratio. J. Biol. Chem..

[68] Takasu Y., Yamada H., Tsubouchi K. (2002). Isolation of three main sericin components from the cocoon of the silkworm, *Bombyx mori*. Biosci. Biotechnol. Biochem..

[69] Zhang Y.-Q., Shen W.-D., Xiang R.-L., Zhuge L.-J., Gao W.-J., Wang W.-B. (2007). Formation of silk fibroin nanoparticles in water-miscible organic solvent and their characterization. J. Nanopart. Res..

[70] Murphy A.R., Kaplan D.L. (2009). Biomedical applications of chemically-modified silk fibroin. J. Mater. Chem..

