# Supplementary material for: Revealing the Influence of the Degumming Process in the Properties of Silk Fibroin Nanoparticles
Source: Polymers (Basel). 2019 Dec 9;11(12):2045. doi: 10.3390/polym11122045 (PMC6960545; doi:10.3390/polym11122045)
Supplement: Supplementary file 1 [file polymers-11-02045-s001.pdf]

polymers

MDPI

Supplementary Information for Article

Revealing the influence of the degumming process in the properties of silk fibroin nanoparticles.

Guzmán Carissimi[1§], A. Abel Lozano-Pérez[2§], Mercedes G. Montalbán[3], Salvador D. Aznar-Cervantes[2], José Luis Cenis[2] and Gloria Víllora[1]*.

[1] Department of Chemical Engineering, Faculty of Chemistry, University of Murcia (UMU), Campus de Espinardo (Murcia), 30100, Spain;

[2] Department of Biotechnology, Instituto Murciano de Investigación y Desarrollo Agrario y Alimentario (IMIDA). La Alberca (Murcia), 30150, Spain.

[3] Department of Chemical Engineering, University of Alicante, Apartado 99, 03080, Alicante, Spain.

§ Authors contributed equally.

* Correspondence: gvillora@um.es; (G.V.)

Received: date; Accepted: date; Published: date

Supplementary Information for Article

2. Materials and Methods

2.5. Morphological, physicochemical and structural characterization of SF fibers and SFNs

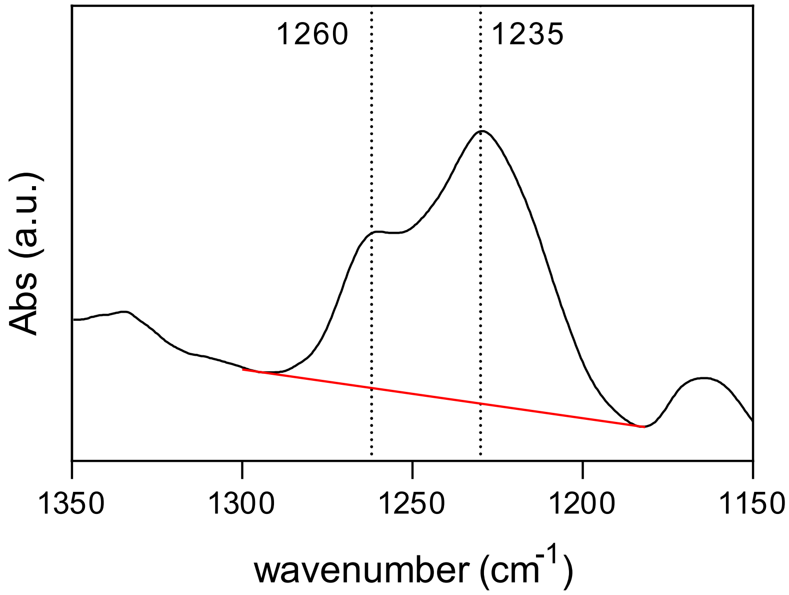

Fig

Figure S1. Example of base line traced in Amide III absorption band for the crystallinity index calculation.

Secondary structure assignation by ATR-FTIR

The secondary structure of the SF samples was also assigned by band fitting of the Amide I region, (1600–1700 cm^{-1}) of the spectra by using OMNIC V9.9.471 software following the approach described by Hu *et al.*[46]. Firstly, a baseline and advanced ATR corrections for one bounce of the incident light at an angle of 45° and a refractive index of 1.60 for the sample were applied to the original spectrum. Then, Fourier Self-Deconvolution (FSD) was applied to the Amide I region as a band narrowing technique. The parameters for the FSD were chosen on the basis of the resolution of the overlapping spectral features. The minimum values of the second derivative of the spectrum obtained by the FSD were used to center the initial bands of Gaussian shapes. Then, the iterative fitting algorithm implemented in the software was used to minimize the residual value between the spectrum and the sum of the deconvoluted bands without baseline.

Table S1. Assignment of the vibration bands in Amide I.

Secondary structure feature	Wavenumber range (cm^{-1})
Aggregate beta-strand/beta-sheets (weak)[a]	1616-1621
β-Sheet (Strong)[a]	1622-1627
β-Sheet (Strong)[b]	1628-1637
Random coil/extended chains	1638-1646
Random coil	1647-1655
α-Helix	1656-1662
Turns	1663-1670
Turns	1671-1685
Turns	1686-1696
β-Sheets (weak)a	1697-1703

[a] Intermolecular β-Sheet; [b] Intramolecular β-Sheets

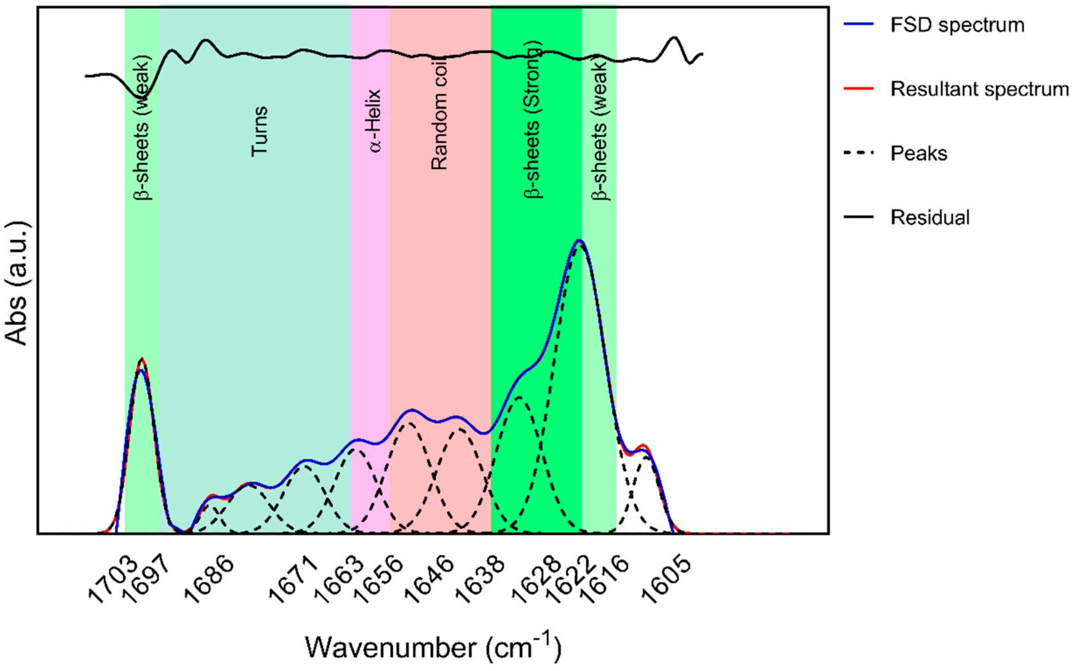

Figure S2. FSD of Amide I absorption band and example of band fitting.

2.5. Morphological, physicochemical and structural characterization of SF fibers and SFNs

Calculation of The surface charge density (σ) of a spherical colloidal particle.

For a nanoparticle with constant surface charge density, the surface charge density is a more characteristic quantity than the Z-potential, ψ_0, because for such particles the Z-potential is not a constant and depends on the electrolyte concentration. The method is based on the following equation:

$$\sigma = \frac{2\varepsilon_r\varepsilon_0\kappa kT}{ze}\sinh\left(\frac{ze\psi_0}{2kT}\right)\left[1+\frac{1}{\kappa a}\frac{1}{cosh^2(ze\psi_0/4kT)}+\frac{1}{(\kappa a)^2}\frac{8\ln[\cosh(\frac{ze\psi_0}{4kT}))}{sinh^2(ze\psi_0/2kT)}\right]^{\frac{1}{2}} \quad (S1)$$

where κ is the Debye-Hückel parameter defined by:

$$\kappa = [\frac{1000e^2N_A(2I)}{\varepsilon kT}]^{1/2} \quad (S2)$$

and k is the Boltzmann constant, T is the absolute temperature, $\mathcal{E}_r$ is relative permittivity of the solvent, $\mathcal{E}_0$ is the permittivity of the vacuum, $\mathcal{E}$ is the permittivity, N_A is the Avogadro's number, e is the elementary electric charge, I is the Ionic strength; a is the radius of the spherical nanoparticles, Ψ_0 is the Z-potential and z is the electrolyte valence. All the parameter values used for the calculation of the surface density charge are shown in the Table S2.

Table S2. List of parameters used to calculate the surface charge density

Parameter	Value	Units
Relative permittivity 25°C, $\mathcal{E}_r$	78,30	*adim.*
Permittivity of a vacuum, $\mathcal{E}_0$	8,85E-12	*C^2/(J·m)*
Permittivity, $\mathcal{E}$	6,93E-10	*C^2/(J·m)*
Boltzmann constant, *k*	1,38E-23	*J/K*
Avogadro's number, N_A	6,02E+23	*mol^{-1}*
Elementary electric charge, e	1,60E-19	*C*
Temperature, *T*	298,15	*K*
Ionic strength, *I*	1,00E-03	*M*
Debye-Hüuckel parameter, κ	1,04E+08	*m^{-1}*
Electrolyte valence, *z*	1	*adim.*

3. Results and Discussion

3.1. Degumming results

Table S3. Degumming efficiency expressed in terms of mass loss and Crystallinity index (CI) of SF samples: D1) Autoclave, D2) Na_2CO_3 30′, D3) Na_2CO_3 120′ and D4) Ultrasounds.

Sample	Mass loss (wt. %)[a]	CI (%)[a]
**SF-D1**	31.3 ± 0.5	59 ± 2
**SF-D2**	32.4 ± 0.8	56 ± 3
**SF-D3**	44.4 ± 1.0	59 ± 2
**SF-D4**	25.9 ± 1.2	52 ± 1

[a]Results are shown as mean ± standard deviation, $n = 3$.

3.2. SF Secondary Structure Analysis

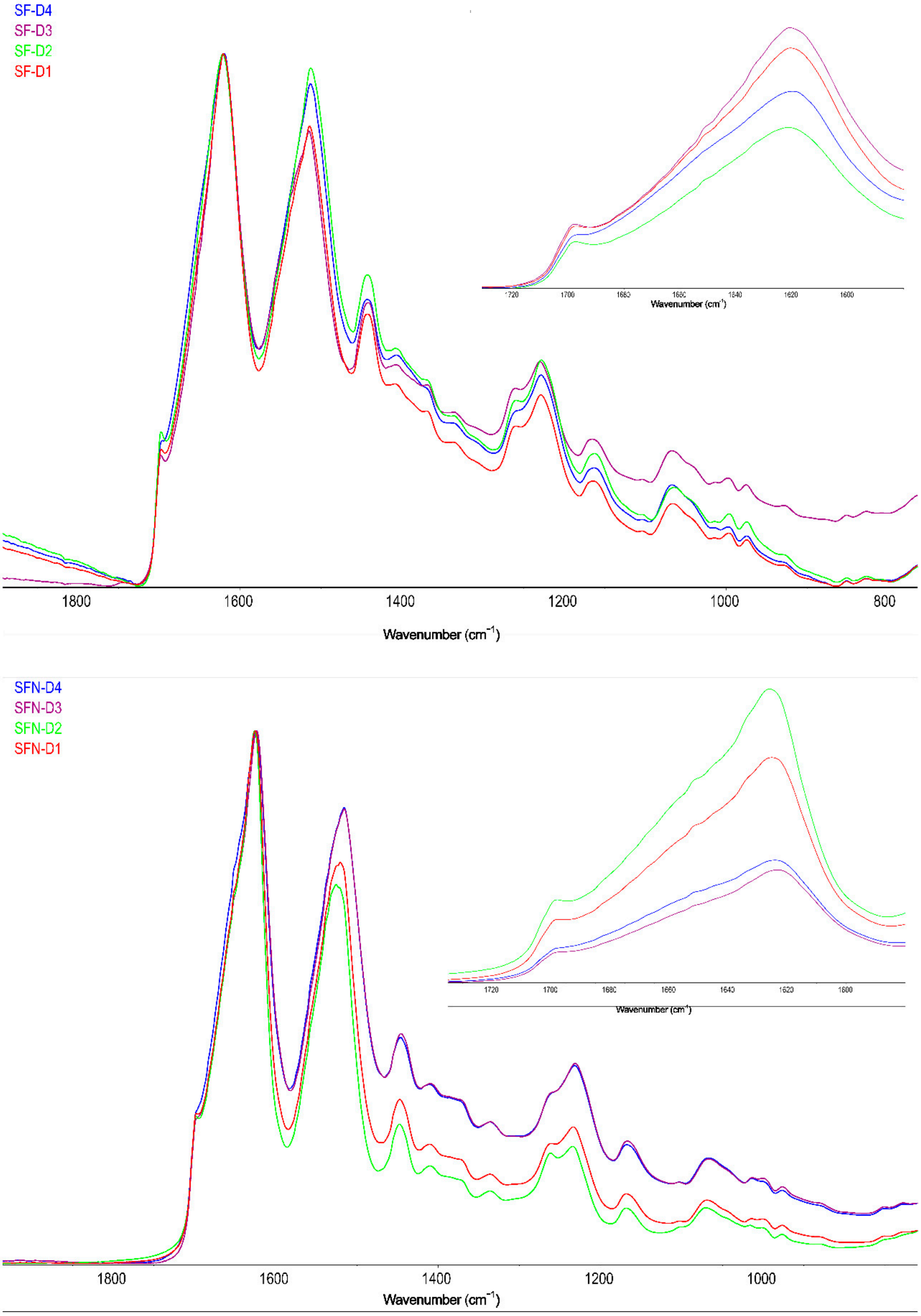

Figure S3. ATR-FTIR spectra of silk fibroins (SF) and silk fibroin nanoparticles (SFN) degummed by: D1) Autoclave, D2) Na_2CO_3 30′, D3) Na_2CO_3 120′ and D4) Ultrasound Amide III absorption band for the crystallinity index calculation. Insets with magnification of the Amide I region of the spectra, used for determination of the secondary structures are included. (Please refer the online version for the color representation of the figure)

Table S4. Relative contribution of secondary structure features of the Amide I in the different stages of the process. SC) Silk Cocoons, internal or external faces, SF) Degummed silk fibroin and SFN) Silk fibroin nanoparticles prepared from SF degummed by: D1) Autoclave, D2) Na_2CO_3 30′, D3) Na_2CO_3 120′ and D4) Ultrasound obtained by FSD analysis of the Amide I infrared absorption band. n = 3, average ± standard deviation.

Sample	β-Sheet	Random Coil	α-Helix	Turn
**SF-D1**	53.6 ± 2.6	21.5 ± 1.0	9.7 ± 0.6	15.2 ± 0.7
**SF-D2**	51.2 ± 1.1	22.4 ± 0.3	9.9 ± 0.4	16.5 ± 0.7
**SF-D3**	50.0 ± 1.0	24.5 ± 1.1	10.0 ± 0.2	15.5 ± 0.7
**SF-D4**	46.7 ± 0.8	21.6 ± 0.1	11.2 ± 0.6	20.5 ± 0.4
**SFN-D1**	59.2 ± 0.6	20.4 ± 0.1	8.2 ± 0.2	12.2 ± 0.5
**SFN-D2**	58.9 ± 1.0	21.3 ± 0.7	8.1 ± 0.3	11.7 ± 0.5
**SFN-D3**	60.2 ± 1.0	20.4 ± 1.0	7.1 ± 0.8	12.2 ± 0.7
**SFN-D4**	55.4 ± 0.7	21.9 ± 0.4	8.4 ± 0.5	14.3 ± 0.8

Table S5. Analysis of variance (ANOVA) of the relative contribution of secondary structure (n = 3).

Tukey's multiple comparisons test	Mean Diff.	95.00% CI of diff.	Significant ?	Adjusted P Value
		**β-Sheet**		
**SF-D1 vs. SF-D2**	2.4	-0.6805 to 5.480	No	0.2661
**SF-D1 vs. SF-D3**	3.6	0.5195 to 6.680	Yes	0.0098
**SF-D1 vs. SF-D4**	6.9	3.820 to 9.980	Yes	<0.0001
**SF-D2 vs. SF-D3**	1.2	-1.880 to 4.280	No	0.9579
**SF-D2 vs. SF-D4**	4.5	1.420 to 7.580	Yes	0.0004
**SF-D3 vs. SF-D4**	3.3	0.2195 to 6.380	Yes	0.026
**SFN-D1 vs. SFN-D2**	0.2837	-2.797 to 3.364	No	>0.9999
**SFN-D1 vs. SFN-D3**	-1.02	-4.101 to 2.060	No	0.9855
**SFN-D1 vs. SFN-D4**	3.784	0.7032 to 6.864	Yes	0.0052
**SFN-D2 vs. SFN-D3**	-1.304	-4.385 to 1.776	No	0.9305
**SFN-D2 vs. SFN-D4**	3.5	0.4195 to 6.580	Yes	0.0137
**SFN-D3 vs. SFN-D4**	4.804	1.724 to 7.885	Yes	0.0001
		**Random Coil**		
**SF-D1 vs. SF-D2**	-0.9	-3.980 to 2.180	No	0.9941
**SF-D1 vs. SF-D3**	-3	-6.080 to 0.08047	No	0.0628
**SF-D1 vs. SF-D4**	-0.1	-3.180 to 2.980	No	>0.9999
**SF-D2 vs. SF-D3**	-2.1	-5.180 to 0.9805	No	0.453
**SF-D2 vs. SF-D4**	0.8	-2.280 to 3.880	No	0.9976
**SF-D3 vs. SF-D4**	2.9	-0.1805 to 5.980	No	0.0824
**SFN-D1 vs. SFN-D2**	-0.8918	-3.972 to 2.189	No	0.9945
**SFN-D1 vs. SFN-D3**	0	-3.080 to 3.080	No	>0.9999
**SFN-D1 vs. SFN-D4**	-1.492	-4.572 to 1.589	No	0.8555
**SFN-D2 vs. SFN-D3**	0.8918	-2.189 to 3.972	No	0.9945
**SFN-D2 vs. SFN-D4**	-0.6	-3.680 to 2.480	No	0.9998
**SFN-D3 vs. SFN-D4**	-1.492	-4.572 to 1.589	No	0.8555
		**α-Helix**		
**SF-D1 vs. SF-D2**	-0.2	-3.280 to 2.880	No	>0.9999
**SF-D1 vs. SF-D3**	-0.3	-3.380 to 2.780	No	>0.9999
**SF-D1 vs. SF-D4**	-1.5	-4.580 to 1.580	No	0.8515
**SF-D2 vs. SF-D3**	-0.1	-3.180 to 2.980	No	>0.9999
**SF-D2 vs. SF-D4**	-1.3	-4.380 to 1.780	No	0.9317
**SF-D3 vs. SF-D4**	-1.2	-4.280 to 1.880	No	0.9579
**SFN-D1 vs. SFN-D2**	0.06327	-3.017 to 3.144	No	>0.9999
**SFN-D1 vs. SFN-D3**	1.02	-2.060 to 4.101	No	0.9855
**SFN-D1 vs. SFN-D4**	-0.2367	-3.317 to 2.844	No	>0.9999
**SFN-D2 vs. SFN-D3**	0.9571	-2.123 to 4.038	No	0.9908
**SFN-D2 vs. SFN-D4**	-0.3	-3.380 to 2.780	No	>0.9999
**SFN-D3 vs. SFN-D4**	-1.257	-4.338 to 1.823	No	0.944

		**β-Turn**		
**SF-D1 vs. SF-D2**	-1.3	-4.380 to 1.780	No	0.9317
**SF-D1 vs. SF-D3**	-0.3	-3.380 to 2.780	No	>0.9999
**SF-D1 vs. SF-D4**	-5.3	-8.380 to -2.220	Yes	<0.0001
**SF-D2 vs. SF-D3**	1	-2.080 to 4.080	No	0.9874
**SF-D2 vs. SF-D4**	-4	-7.080 to -0.9195	Yes	0.0024
**SF-D3 vs. SF-D4**	-5	-8.080 to -1.920	Yes	<0.0001
**SFN-D1 vs. SFN-D2**	0.5449	-2.536 to 3.625	No	0.9999
**SFN-D1 vs. SFN-D3**	7.105e-15	-3.080 to 3.080	No	>0.9999
**SFN-D1 vs. SFN-D4**	-2.055	-5.136 to 1.025	No	0.4846
**SFN-D2 vs. SFN-D3**	-0.5449	-3.625 to 2.536	No	0.9999
**SFN-D2 vs. SFN-D4**	-2.6	-5.680 to 0.4805	No	0.1733
**SFN-D3 vs. SFN-D4**	-2.055	-5.136 to 1.025	No	0.4846
